# Tobacco-induced melanosis on the dorsal surface of the tongue: a rare clinical image

**DOI:** 10.11604/pamj.2022.43.49.37112

**Published:** 2022-09-30

**Authors:** Nidhi Adyalkar, Neha Shetty

**Affiliations:** 1 Department of Research and Development, Jawaharlal Nehru Medical College, Datta Meghe Institute of Medical Sciences, Sawangi (Meghe), Wardha, India,; 2 Department of Oral and Maxillofacial Surgery, Sharad Pawar Dental College, Datta Meghe Institute of Medical Sciences, Sawangi (Meghe), Wardha, India

**Keywords:** Tongue, melanosis, pigmentation, tobacco

## Image in medicine

**Figure 1:**
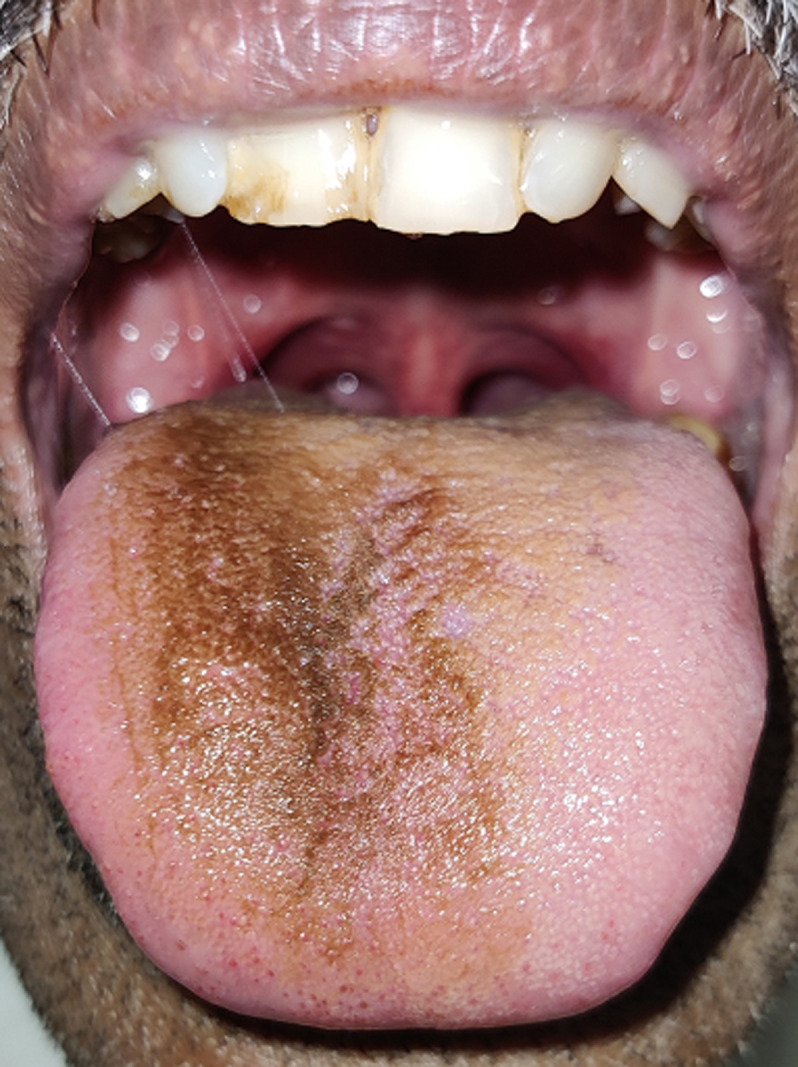
tobacco-induced melanosis on dorsal surface of the tongue

The use of smoking and smokeless tobacco causes pigmentation on the oral mucosal as mucosal pigmentation, which is one of the clinical features of tobacco-induced melanosis. Tobacco-induced pigmentation is seen on the tongue of the presented case (Figure 1). He had a habit of kharra chewing 3 times a day since 15 years, kharra is composed of tobacco, betelnut and slaked lime. Tobacco is the main content of kharra and is responsible for the pigmentation of the oral mucosa. Accumulation of melanin pigment in tissue causes melanosis. Melanosis increases with the increasing exposure of tobacco to the oral mucosa and associated physiologic melanosis. In presenting case patient had congenital melanoma and the habit of quid placement on the tongue. There is no treatment for tobacco-induced melanosis. Tobacco induced melanosis can convert to normal mucosal colour within 6 to 36 months if the patient withdraws kharra chewing habit.

